# Efficacy of a stannous fluoride dentifrice for relieving dentinal hypersensitivity in Chinese population: an 8-week randomized clinical trial

**DOI:** 10.1007/s00784-024-05610-9

**Published:** 2024-03-26

**Authors:** Rui Li, Wenjie Yang, Roberta Grimaldi, Peter Zeng, Gary Smith, Xi Chen

**Affiliations:** 1 Department of Preventive Dentistry, Shanghai Ninth People’s Hospital, No. 639 Zhizaoju Road, Shanghai, China; 2 Shanghai Jiao Tong University School of Medicine, Shanghai, China; 3 College of Stomatology, Shanghai Jiao Tong University, Shanghai, China; 4 National Center for Stomatology, Shanghai, China; 5 National Clinical Research Center for Oral Diseases, Shanghai, China; 6 Shanghai Key Laboratory of Stomatology, Shanghai, China; 7 Shanghai Research Institute of Stomatology, Shanghai, China; 8 Haleon (Formerly Known As GSK Consumer Healthcare), Weybridge, UK; 9 Haleon (Formerly Known As GSK Consumer Healthcare), Shanghai, China

**Keywords:** Dentinal hypersensitivity, Stannous fluoride, Dentifrice, Schiff sensitivity score, Tactile threshold, Placebo effect

## Abstract

**Objectives:**

To compare the effectiveness of using a 0.454% stannous fluoride-containing dentifrice twice daily in relieving dentinal hypersensitivity (DH) in a Chinese population.

**Materials and methods:**

This was a single-centre, randomized, controlled, examiner-blind, three-treatment-arm, parallel-group study in participants with clinically diagnosed DH. Subjects who met inclusion criteria (n = 197) were randomly allocated into 3 groups: test group (n = 66)—using a 0.454% stannous fluoride-containing dentifrice twice daily; positive control group (n = 64)—using a 5.0% calcium sodium phosphosilicate-containing dentifrice twice daily; negative control group (n = 67)—using a 1150 ppm of NaF dentifrice twice daily. Status of DH was assessed at week 4 and week 8 by the same independent examiner. Changes from baseline in Schiff sensitivity score, tactile threshold and Dentine Hypersensitivity Experience Questionnaire (DHEQ) score were analysed using analysis of covariance models. The DHEQ evaluated subject-perceived oral health-related quality of life (OHRQoL).

**Results:**

Statistically significant improvements in mean Schiff scores (p < 0.0001 for all products at Weeks 4 and 8), tactile threshold (p < 0.0001 for test and negative control at Weeks 4 and 8: Week 4 p = 0.0040 and Week 8 p < 0.0001 for positive control) and all DHEQ scores (p < 0.01 for all groups) were observed. No statistically significant differences were observed in the adjusted mean between all dentifrices at both timepoints, potentially driven by a placebo effect. Forty-two treatment-emergent adverse events (TEAEs) were reported (n = 32 subjects), with 2 serious AEs (n = 1) in the test group. TEAEs were not considered treatment-related.

**Conclusions:**

All groups demonstrated statistically significant improvements in Schiff score, tactile threshold and OHRQoL. However, due to a possible placebo effect in the negative control, there were no statistically significant differences between the dentifrices.

**Clinical relevance:**

This study adds to the growing research exploring why the placebo effect is a common phenomenon in DH studies.

**Trial registration:** ClinicalTrials.gov Identifier: NCT04950465.

**Supplementary Information:**

The online version contains supplementary material available at 10.1007/s00784-024-05610-9.

## Introduction

Dentinal hypersensitivity (DH) is a common global oral health issue [1, 2]. This condition is characterized by a short, sharp pain or sensitivity when the dentin is exposed to thermal, tactile, osmotic, chemical, or evaporative stimuli [1, 2]. The primary aetiological factors associated with the onset of DH include gingival recession and/or enamel loss (e.g. through erosion or abrasion), which can result in exposure of dentine with patent dentinal tubules [2, 3]. The hydrodynamic theory of DH hypothesizes that an external stimulus to the tooth (e.g. a temperature/osmotic differential) causes movement of the fluid resident within dentinal tubules [4]. This fluid movement may stimulate nerve processes in the pulpal area of the dentine, including irritation of odontoblasts, pulpal neurons, and even subodontoblastic blood vessels, resulting in the characteristic short, sharp pain of DH [2, 5, 6].

Currently, there are two approaches to the management of DH: nerve depolarization or dentine tubule occlusion [7, 8]. Nerve depolarizing agents – typically potassium salts – generally require a period of use (e.g. 2–4 weeks) before their benefit is established [3, 8, 9]. The delivery of potassium ions to the dentine-pulp junction (odontoblastic layer) via exposed dentine tubules is believed to result in depolarization of the afferent nerve membrane, thereby interrupting the pain response [3, 8, 9]. The second approach includes the use of occluding agents (e.g. stannous fluoride [SnF_2_]), which act by physically blocking or narrowing the exposed end of dentinal tubules (by the precipitation of insoluble materials onto the dentine surface and/or within dentinal tubules), thus reducing disruption to dentinal fluid movement in response to an external stimulus [2, 10–12].

Previous randomized controlled trials have shown the efficacy of dentifrices containing 0.454% weight by weight (w/w) SnF_2_ for the relief of DH measured by Schiff sensitivity score, tactile threshold or visual analogue score. Statistically significant reductions in pain and sensitivity were seen with the 0.454% w/w SnF_2_ product compared with control dentifrices without 0.454% w/w SnF_2_, with relief of DH observed in subjects during short- and long-term use [13–17]. However, in Chinese population, subjects used dentifrices containing 0.454% w/w SnF_2_ twice daily showed no significant advantage over negative or positive controls[18].

Furthermore, a participant-reported outcome study also demonstrated the positive impact of SnF_2_-containing toothpaste in oral health-related quality of life (OHRQoL) [19]. DH may have a potentially significant impact on general quality of life in DH sufferers in terms of an individual’s ability to sleep, drink, eat, brush their teeth, and engage in social interactions [19–23]. OHRQoL questionnaires are tools increasingly used in dentistry to capture the impact of clinical interventions on OHRQoL [24]. The Dentine Hypersensitivity Experience Questionnaire (DHEQ) is a validated, condition-specific measure of OHRQoL in relation to DH [25, 26]. The short-form of the questionnaire (DHEQ-15) was used in this study and includes questions related to individuals’ daily activities, frequency of teeth sensations, and the impact this may have on overall quality of life [27, 28]. Further research has also shown the DHEQ-15 has high internal reliability and validity for assessment of patients with DH in China [29].

The primary objective of this study was to compare the clinical efficacy of 8 weeks’ twice-daily brushing with a 0.454% w/w SnF_2_ dentifrice (test) against a commercially available regular fluoride (negative control) in a Chinese population diagnosed with DH, as measured by response to evaporative (air) stimulus (Schiff sensitivity score). A clinically proven positive control was included in this study as a benchmark of performance previously seen in a Chinese population [30, 31].

The secondary objective was a comparison of response to a tactile stimulus (Yeaple probe) at Weeks 4 and 8 compared with evaporative (air) stimulus (Schiff sensitivity score) at Week 4 (test vs. negative control, and vs. positive control). Exploratory objectives included the characterization of the OHRQoL, as measured by DHEQ-15, after 8 weeks of treatment with the test and positive control dentifrice compared against the negative control dentifrice.

## Materials and methods

### Study design

This was a single-centre, randomized, controlled, examiner-blind, three-treatment-arm, parallel-group design study in healthy participants with self-reported and clinically assessed DH, with at least two sensitive teeth (NCT04950465). An examiner-blind design was selected to minimize bias and maximize the validity of the results. The study was conducted in Shanghai Ninth People’s Hospital (Shanghai, China), in line with published recommendations and the requirement of Chinese Ministry of Health guidelines [32, 33]. It was performed in full compliance with the International Council for Harmonization of Technical Requirements for Registration of Pharmaceuticals for Human Use and all applicable local Good Clinical Practice regulations.

### Study population

Subjects recruited for this study were males and females aged 18–70 years, who had good physical and mental health, and who were able to comply with study procedures, treatment plan and scheduled visits. Subjects also had a history of DH > 6 months but not more than 10 years, with ≥ 20 natural, permanent teeth and a minimum of two accessible non-adjacent teeth (incisors, canines, and pre-molars) that met the key inclusion criteria, as assessed by the investigator or a medically qualified designee. Subjects also had to own a smartphone with the WeChat application installed for the duration of the study to receive reminders regarding brushing schedules and further communications.

Key inclusion criteria included signs of facial/cervical gingival recession and/or signs of erosion and abrasion; teeth with a modified gingival index score ≤ 1 adjacent to the two test teeth with exposed dentine; a clinical mobility ≤ 1; and teeth with signs of sensitivity using a tactile stimulus (Yeaple ≤ 20 g) and an evaporative (air) stimulus (Schiff sensitivity score ≥ 2). All inclusion criteria were confirmed at the baseline examination (Visit 2).

Subjects were excluded if they used another sensitivity relief toothpaste or product within 30 days of the first dose of the study test product. Additional exclusion criteria included subject participation in another clinical trial 30 days prior to study entry or participation; smoking; a history of regular alcohol or substance use; pregnancy; breastfeeding; intolerance or hypersensitivity to the study product ingredients; a tongue or lip piercing; use of antibiotics within 2 weeks of screening and/or baseline; use of medication (in the opinion of the investigator) causing xerostomia; or use of medications, treatments or herbal ingredients that could interfere with the perception of pain.

Specific dental exclusion criteria for this study included subject participation in another tooth desensitizing treatment study within 8 weeks of the screening visit; dental prophylaxis within 4 weeks of screening, or antibiotic prophylaxis required for dental procedures; advanced periodontal disease; treatment of periodontal disease (including surgery) within 12 months of screening, scaling or root planing within 3 months of screening; teeth bleaching within 8 weeks of screening; rinsing with water during the first minute of toothbrushing at the screening visit; exposed tooth dentine with deep, defective or facial restorations; teeth used as abutments for fixed or removable partial dentures; teeth with full crowns or veneers, orthodontic bands or cracked enamel; or sensitive teeth arising from aetiologies other than erosion, abrasion or recession of exposed dentine.

### Study procedures

At screening (Visit 1), study information was explained to subjects, and they gave written, informed consent prior to any study procedures taking place. Subjects were requested by clinical study site staff to bring their current oral care products to the study site to confirm the dentifrice ingredients did not impact sensitivity relief, which could affect the study results. Additionally, at Visit 1, subjects were asked to demonstrate their typical daily oral care regimen to make sure they did not rinse while brushing. Qualifying tactile (Yeaple probe) and evaporative (air) sensitivity assessments (Schiff sensitivity scores) on the incisor, canine and pre-molar teeth were conducted by appropriately trained examiners (one examiner per each assessment). Eligible subjects identified at screening (Visit 1) were provided with a soft bristle toothbrush (Lion Thin Bristle, Chinese marketplace) and a regular fluoride toothpaste (Crest Cavity Protection Fresh Lime®; Chinese marketplace; 1150 parts per million (ppm) of sodium fluoride—NaF) and entered a 2–4-week acclimatization period to standardize oral hygiene and become familiar with the study activities.

At baseline (Visit 2), approximately 2–4 weeks after screening (Visit 1), compliance was assessed and full oral soft tissue examination was conducted by an appropriately trained clinical examiner. Eligible teeth were re-assessed and the two non-adjacent (Where possible, from different quadrants) test teeth were selected from those teeth eligible for both Schiff (Schiff ≥ 2) and tactile (≤ 20 g) assessment criteria at screening and baseline. Eligible subjects were then stratified by maximum baseline Schiff sensitivity score (of the two selected test teeth, with a score of 2 or 3) to ensure balance across the treatment groups; they were also provided with a new soft bristle toothbrush and randomized to one of the three treatment groups: the 0.454% w/w SnF_2_ test product (Sensodyne Sensitivity and Gum®); the positive control dentifrice containing 5.0% calcium sodium phosphosilicate (Sensodyne Repair and Protect®); or the negative control dentifrice containing 1150 ppm of NaF (Crest Cavity Protection Fresh Lime®). All products were commercially available in the Chinese marketplace.

Subjects were asked to apply a ribbon of their assigned toothpaste to cover the head of the toothbrush, and to brush their teeth in their usual manner for 1 timed minute twice daily, every morning and evening, and rinse with 10 ml water after brushing. Subjects randomized to the test product were also asked to focus brushing on the two test teeth as per commercial label instructions. First use was carried out under supervision at the study site and then brushing at home by themselves. In order to improve the subjects' compliance with brushing their teeth, each subject was given a separate diary card to record daily brushing and the diary card was brought back for checking by researcher at each visit. Subjects returned to site after 4 and 8 weeks, and the toothbrush was replaced at Week 4. Following toothbrushing and rinsing, subjects could also conduct a discretionary tongue clean using the provided toothbrush, but this was not a study requirement. Compliance (Supplemental methods) and adverse events (AEs) were checked throughout the study.

Sensitivity of the two test teeth was evaluated by an evaporative (air) stimulus (Schiff sensitivity score) and a tactile stimulus (Yeaple probe) at baseline, Week 4 and Week 8. Participants were asked to refrain from eating and drinking for at least 2 h prior to study visits, except for small sips of room-temperature water up to 1 h prior to assessments. Participants were to refrain from excessive alcohol consumption for 24 h prior to baseline, Week 4 and Week 8 visits.

As part of the exploratory outcome, subjects also completed the short-form version of the validated OHRQoL questionnaire (DHEQ-15) at baseline and Week 8.

### Sensitivity assessments

The tactile sensitivity of incisor, canine and pre-molar teeth was assessed with a constant pressure probe (Yeaple probe) placed perpendicular to the facial surface of the tooth and drawn slowly across the exposed dentine to ensure application of the stimulus across the potentially ‘sensitive’ area [34, 35]. After each application, subjects were asked to indicate whether the sensation caused pain or discomfort. Only ‘yes’ and ‘no’ were acceptable answers. If no pain response was found, the tactile threshold was recorded as > 20 g and the tooth was disqualified from further assessment.

The evaporative (air) sensitivity assessments were assessed a minimum of 5 min after the tactile assessments have been completed [35, 36]. Subject’s response was categorized into 4-point Schiff sensitivity scores (0 defined as a subject who did not respond to air stimulation; 1 defined as a subject who responded to the air stimulus but did not request discontinuation of the stimulus; 2 defined as a subject who responded to the air stimulus and requested discontinuation or moved away from the stimulus; and 3 defined as a subject who responded to the stimulus, considered the stimulus to be painful and also requested discontinuation of the stimulus). In order for a tooth to qualify and potentially be selected as the ‘test tooth’, it must have had a tactile threshold ≤ 20 g and a Schiff sensitivity score ≥ 2 at screening and baseline.

If any of the test teeth identified at screening did not respond to the tactile stimulus at baseline, all eligible teeth that qualified at screening were re-assessed for tactile threshold and Schiff sensitivity score until two new ‘test teeth’ were identified. At Visits 3 and 4, the tactile and air evaporative assessments were conducted on the two selected ‘test teeth’ only.

### Statistical methods

This study planned to screen an adequate number of subjects to randomize ~ 195 subjects, to ensure ~ 180 subjects completed the study (assuming a 10% dropout). This allowed for a 90% power to detect a mean difference (standard deviation [SD]) of 0.3 (0.501) in the change from baseline in Schiff sensitivity score after 8 weeks of treatment, representing ~ 15% difference between treatment groups. A modified intent-to-treat population (mITT) was used for efficacy analyses, comprising all randomized participants who had a post-baseline assessment.

The Schiff sensitivity score and tactile threshold score at each visit were derived as the average of the two test teeth (Supplemental methods). The primary study endpoint compared the change from baseline in Schiff sensitivity score at Week 8 between the negative control and test dentifrice. Secondary study endpoints compared the change from baseline in tactile threshold at Weeks 4 and 8 (test product vs. negative control), the change from baseline in Schiff sensitivity score at Week 4 (test product vs. negative control), and the change from baseline in Schiff sensitivity score and tactile threshold at Weeks 4 and 8 (positive control vs. negative control).

The exploratory endpoint compared the change from baseline at Week 8 for the test and positive control dentifrice against the negative control dentifrice in responses to questions 7–9 for DHEQ-15 Sect. 1, the total score (questions 1–15) for DHEQ-15 Sect. 2, and the restrictions, adaptation, social impact, emotional impact and identity domain scores for DHEQ-15 Sect. 2. Lastly, the safety endpoints assessed the number of AEs.

The change from baseline in Schiff sensitivity score, tactile threshold, and DHEQ-15 scores was analysed using analysis of covariance (ANCOVA) models with the study product as a factor and baseline score as a covariate. For the tactile threshold and DHEQ-15 ANCOVAs, an additional factor for the baseline Schiff stratification was included. Due to non-normally distributed residuals from the tactile threshold and DHEQ-15 ANCOVAs, the products were compared using a van Elteren test stratified by baseline Schiff stratification. Significance testing was conducted at the two-sided 5% significance level; no adjustments for multiple comparisons were made for secondary endpoints.

Following analysis of Schiff sensitivity score and tactile threshold, an unexpected change from baseline was observed in the negative control dentifrice group (p < 0.0001 at Weeks 4 and 8). Therefore, a post-hoc analysis was conducted to explore the potential factors that might have caused the differences in the treatment groups, to better understand the study results. The effect of an extended acclimatization period due to study site closure resultant from Covid-19 outbreak was investigated across the three treatment groups, as were fluctuations in DH symptoms between screening and baseline on Schiff and tactile study results. Subjects were assessed within the following subgroups:

- 1a. Subjects with stable DH (defined as subjects with no changes in Schiff sensitivity score between screening and baseline [from 2 to 2, or 3 to 3])
- 1b. Subjects with non-stable DH (defined as subjects with changes in Schiff sensitivity score between screening and baseline [from 2 to 3, or 3 to 2])
- 2a. Subjects without extended acclimatization period (up to Week 4 as per protocol)
- 2b. Subjects with extended acclimatization period (up to 2.5 months due to study site closure)
- 3. Subjects with both stable DH and without extended acclimatization period

## Results

### Study population

**Fig. 1:**
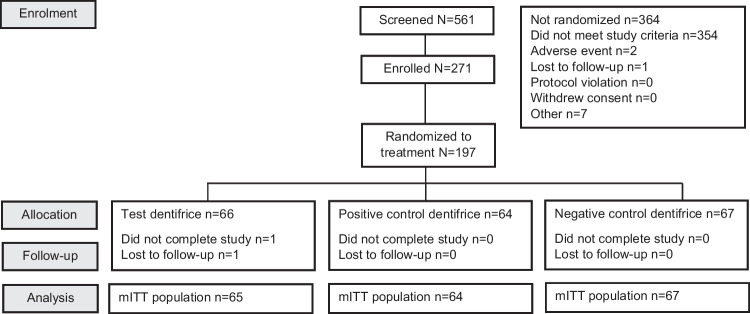
Study disposition in the mITT population. Test = Sensodyne Sensitivity and Gum®; Negative control = Crest Cavity Protection®; Positive control = Sensodyne Repair and Protect®. mITT = modified intent-to-treat; N/n = number of subjects

The disposition of the study population is presented in Fig. 1. A total of 561 subjects were screened, 271 were enrolled and 197 were randomized across the three treatment groups. A total of 196 (99.5%) subjects completed the study, with 66 subjects in the test product group (1 dropped during follow-up), 64 subjects in the positive control dentifrice group, and 67 subjects in the negative control dentifrice group.

Baseline demographics and characteristics are reported in Table 1. The majority of the study population were female (90.3%), and all (100%) were of Asian – East Asian heritage. The mean (± SD) age of the study population was 43.0 (± 8.76) years, with a range of 20–69 years. Subjects were stratified at the baseline visit according to their maximum Schiff sensitivity score of 2 (40.8%) and 3 (59.2%).

**Table 1:** Baseline demographics and characteristics in the mITT population

	Test (n = 65)	Negative control (n = 67)	Positive control (n = 64)	Overall (N = 196)
Sex, n (%)				
Male	8 (12.3)	3 (4.5)	8 (12.5)	19 (9.7)
Female	57 (87.7)	64 (95.5)	56 (87.5)	177 (90.3)
Race, n (%)				
Asian – East Asian heritage^a^	65 (100)	67 (100)	64 (100)	196 (100)
Ethnicity, n (%)				
Hispanic or Latino	0	1 (1.5)	0	1 (0.5)
Not Hispanic or Latino	65 (100)	66 (98.5)	64 (100)	195 (99.5)
Age (years)				
n	65	67	64	196
Mean (SD)	43.9 (8.04)	42.7 (8.55)	42.4 (9.68)	43.0 (8.76)
Stratification, n (%)				
Maximum baseline Schiff score 2	25 (38.5)	29 (43.3)	26 (40.6)	80 (40.8)
Maximum baseline Schiff score 3	40 (61.5)	38 (56.7)	38 (59.4)	116 (59.2)

*mITT* modified intent-to-treat, *n* number of observations, *SD* standard deviation

^a^Study population only included participants of East Asian heritage, therefore no other races are shown. Test = Sensodyne Sensitivity and Gum®; Negative control = Crest Cavity Protection®; Positive control = Sensodyne Repair and Protect®

### Efficacy

#### Schiff sensitivity score

Mean Schiff sensitivity scores are reported in Table 2. A statistically significant reduction from baseline (p < 0.0001) in the Schiff sensitivity score was observed at Weeks 4 and 8 in the test product, positive control dentifrice and negative control dentifrice groups. There was no statistically significant difference in the change from baseline in Schiff sensitivity score between the test product and negative control at Week 4 (p = 0.7645) and Week 8 (primary endpoint, p = 0.2639). Similar findings were observed when comparing the positive control and negative control at Week 4 (p = 0.9728) and Week 8 (p = 0.2968).

**Table 2:** Statistical analysis of change from baseline in Schiff sensitivity score over time in the mITT population

			Change from baseline				Comparison with negative control			
Visit	Study product	n	Baseline Schiff score (mean ± SD)	Adjusted mean (SE)^a^	95% CI^a^	p-value^a^	Adjusted mean difference (SE)^a^	Adjusted mean difference (%)	95% CI^a^	p-value^a^
Week 4	Test	64	2.50 ± 0.442	–0.79 (0.082)	–0.95, –0.63	< 0.0001	–0.03 (0.115)	4.6	–0.26, 0.19	0.7645
	Positive control	64	2.47 ± 0.435	–0.76 (0.082)	–0.92, –0.60	< 0.0001	–0.00 (0.1114)	0.5	–0.23, 0.22	0.9728
	Negative control	67	2.43 ± 0.425	–0.75 (0.080)	–0.91, –0.60	< 0.0001				
Week 8	Test^b^	65		–0.98 (0.099)	–1.18, –0.79	< 0.0001	0.16 (0.140)	–13.7	–0.12, 0.43	0.2639
	Positive control	64		–0.99 (0.100)	–1.19, –0.79	< 0.0001	0.15 (0.140)	–12.9	–0.13, 0.42	0.2968
	Negative control	67		–1.14 (0.098)	–1.33, –0.94	< 0.0001				

*ANCOVA* analysis of covariance, *CI* confidence interval, *mITT* modified intent-to-treat, *n* number of observations, *SD* standard deviation, *SE* standard error

^a^Analysis was performed using ANCOVA model with study product as a factor and baseline Schiff sensitivity score as a covariate. Positive % adjusted mean difference favours test/positive control

^b^Primary endpoint. Test = Sensodyne Sensitivity and Gum®; Negative control = Crest Cavity Protection®; Positive control = Sensodyne Repair and Protect®

#### Tactile threshold score

Mean tactile threshold (g) scores are reported in Table 3. A statistically significant increase from baseline in the tactile threshold (g) was observed at Weeks 4 and 8 (p < 0.0001) in the test dentifrice and negative control dentifrice group and at both timepoints in the positive control dentifrice (Week 4 p = 0.0040; Week 8 p < 0.0001). There was no statistically significant difference in the change from baseline in tactile threshold score between the test product vs. the negative control (Week 4 p = 0.6978; Week 8 p = 0.8130) and the positive control vs. negative control (Week 4 p = 0.1682; Week 8 p = 0.3937).

**Table 3:** Statistical analysis of change from baseline in tactile threshold (g) over time in the mITT population

			Change from baseline				Comparison with negative control			
Visit	Study product	n	Baseline tactile threshold (mean ± SD)	Adjusted mean (SE)^a^	95% CI^a^	p-value^a^	Adjusted mean difference (SE)^a^	% Adjusted mean difference	95% CI^a^	p-value^b^
Week 4	Test	64	11.5 ± 2.33	9.22 (1.577)	6.11, 12.33	< 0.0001	1.80 (2.204)	24.3	–2.54, 6.16	0.6978
	Positive control	64	12.1 ± 2.79	4.59 (1.577)	1.48, 7.70	0.0040	–2.82 (2.203)	–38.1	–7.17, 1.52	0.1682
	Negative control	67	11.8 ± 2.71	7.42 (1.538)	4.38, 10.45	< 0.0001				
Week 8	Test	65		16.63 (2.393)	11.91, 21.35	< 0.0001	4.81 (3.356)	40.7	–1.81, 11.43	0.8130
	Positive control	64		11.05 (2.412)	6.30, 15.81	< 0.0001	–0.76 (3.370)	–6.4	–7.41, 5.89	0.3937
	Negative control	67		11.82 (2.353)	7.18, 16.46	< 0.0001				

*Test* Sensodyne Sensitivity and Gum®, *Negative control* Crest Cavity Protection®, *Positive control* Sensodyne Repair and Protect®

*ANCOVA* analysis of covariance, *CI* confidence interval, *mITT* modified intent-to-treat, *n* number of observations, *SD* standard deviation, *SE* standard error

^a^Analysis was performed using ANCOVA model with study product and baseline Schiff stratification as factors and baseline tactile threshold as a covariate

^b^P-value from van Elteren test. Positive % adjusted mean difference favours test/positive control

#### DHEQ-15 responses

The summary of subject responses to the DHEQ-15 Sect. 1, questions 1–6 at baseline are presented in Table S1. Statistical analysis of change from baseline in response to the DHEQ-15 Sect. 1, questions 7–9 at Week 8 are presented in Table S2. A statistically significant reduction in the mean score from baseline to Week 8 was observed for questions 7, 8, and 9 in the product groups (p < 0.0001). There were no statistically significant differences between treatment groups for DHEQ-15 Sect. 1, questions 7–9 test product vs. negative control dentifrice for question 7 (p = 0.1398), question 8 (p = 0.2693) and question 9 (p = 0.0743), and positive control dentifrice vs. negative control dentifrice for question 7 (p = 0.1456), question 8 (p = 0.3209) and question 9 (p = 0.3759) (Table S2).

Statistical analysis of change from baseline in response to the DHEQ-15 Sect. 2, questions 1–15 total score for all domains and each domain at Week 8 are presented in Table 4. A statistically significant reduction from baseline in the mean DHEQ-15 total score at Week 8 was observed in the test product (p < 0.0001), positive control (p < 0.0001) and negative control (p = 0.0002) dentifrice groups. Significant reductions (p < 0.01) were also observed across all mean domain scores (restrictions, adaptation, social impact, emotional impact, and identity) from baseline to Week 8 in the test product, positive control, and negative control dentifrice groups.

**Table 4:** Statistical analysis of change from baseline in response to the DHEQ Sect. 2, (Q1–Q15) total score for all domains and each domain at Week 8 in the mITT population

Domain	Visit	Study product	n	Adjusted mean (SE)^a^	95% CI^a^	p-value^a^	Adjusted mean difference (SE)^a^	Adjusted mean difference (%)	95% CI^a^	p-value^b^
Total score	Week 8	Test	65	–11.28 (2.238)	–15.69, –6.86	< 0.0001	–2.96 (3.140)	35.6	–9.15, 3.23	0.1188
		Positive control	64	–11.30 (2.256)	–15.75, –6.85	< 0.0001	–2.98 (3.153)	35.9	–9.20, 3.24	0.4759
		Negative control	67	–8.32 (2.202)	–12.66, –3.98	0.0002				
Restrictions domain (Q1–Q3)	Week 8	Test	65	–2.03 (0.468)	–2.96, –1.11	< 0.0001	-0.43 (0.658)	27.0	–1.73, 0.87	0.2667
		Positive control	64	–1.81 (0.472)	–2.74, –0.88	0.0002	-0.21 (0.660)	12.9	–1.51, 1.10	0.9649
		Negative control	67	–-1.60 (0.461)	–2.51, –0.69	0.0006				
Adaptation domain (Q4–Q6)	Week 8	Test	65	–2.48 (0.474)	–3.41, –1.54	< 0.0001	–0.75 (0.665)	43.5	–2.06, 0.56	0.2712
		Positive control	64	–2.54 (0.478)	–3.48, –1.60	< 0.0001	–0.81 (0.668)	47.2	–2.13, 0.50	0.3270
		Negative control	67	–1.72 (0.466)	–2.65, –0.80	0.0003				
Social impact domain (Q7–Q9)	Week 8	Test	65	–2.32 (0.485)	–3.28, –1.36	< 0.0001	–0.62 (0.680)	36.8	–1.97, 0.72	0.1997
		Positive control	64	–2.24 (0.489)	–3.21, –1.28	< 0.0001	–0.55 (0.682)	32.3	–1.89, 0.80	0.7862
		Negative control	67	–1.70 (0.476)	–2.64, –0.76	0.0005				
Emotional impact domain (Q10–Q12)	Week 8	Test	65	–2.17 (0.481)	–3.12, –1.22	< 0.0001	–0.61 (0.675)	39.4	–1.95, 0.72	0.2292
		Positive control	64	–2.13 (0.486)	–3.09, –1.18	< 0.0001	–0.58 (0.680)	37.1	–1.92, 0.76	0.8382
		Negative control	67	–1.56 (0.474)	–2.49, –0.62	0.0012				
Identity domain (Q13–Q15)	Week 8	Test	65	–2.25 (0.521)	–3.28, –1.22	< 0.0001	–0.53 (0.731)	30.6	–1.97, 0.92	0.2284
		Positive control	64	–2.63 (0.524)	–3.66, –1.60	< 0.0001	–0.91 (0.733)	52.6	–2.35, 0.54	0.3146
		Negative control	67	–1.72 (0.513)	–2.73, –0.71	0.0009				

^a^Analysis is performed using ANCOVA model with study product and baseline Schiff stratification as factors and baseline score of relevant parameter included as a covariate. ^b^P-value from van Elteren test

Adjusted mean difference is calculated as test/positive control minus negative control such that a negative difference favours test/positive control

Adjusted mean difference (%) is calculated as 100 x (adjusted mean difference/adjusted mean of negative control)

Positive % adjusted mean difference favours test/positive control

All five functional domains (restrictions, adaptations, social impact, emotional impact and identity) have domain scores ranging from 3–21

Total score is for all 15 questions and has a range of 15–105

*Test* Sensodyne Sensitivity and Gum®, *Negative control* Crest Cavity Protection®, *Positive control* Sensodyne Repair and Protect®

*CI* confidence interval, *DHEQ* dentine hypersensitivity experience questionnaire, *mITT* modified intent-to-treat, *n* number of observations, *SE* standard error

There were no statistically significant differences between treatment groups in the total score (DHEQ-15 Sect. 2, questions 1–15) for the test product vs. negative control dentifrice (p = 0.1188) and the positive control dentifrice vs. negative control dentifrice (p = 0.4759) (Table 4). There were also no statistically significant differences between treatment groups observed for DHEQ-15 domains: test product vs. negative control dentifrice (restrictions [p = 0.2667], adaptation [p = 0.2712], social impact [p = 0.1997], emotional impact [p = 0.2292], and identity [p = 0.2284]) and positive control dentifrice vs. negative control dentifrice (restrictions [p = 0.9649], adaptation [p = 0.3270], social impact [p = 0.7862], emotional impact [p = 0.8382], and identity [p = 0.3146]) (Table 4).

### Post-hoc analysis

There were notable differences in the mean Schiff sensitivity score at baseline between the five subgroups (1a, 1b, 2a, 2b and 3) for each treatment group. Subjects with non-stable DH condition (1b) had a notably higher mean baseline Schiff sensitivity score compared with those with stable DH condition (1a), with no notable differences observed at baseline in the tactile threshold (g) between the five subgroups. Overall, greater mean changes from baseline were observed in subjects with non-stable DH condition (1b) and/or with an extended acclimatization period (2b). The difference was most notable in the negative control group when considering non-stable DH condition (1b mean = –1.42) vs. stable DH condition (1a mean = –0.74) and subjects with an extended acclimatization period (2b mean = –1.40) compared with subjects without an extended acclimatization period (2a mean = –0.95). In the subgroup with stable DH (1a) and without extended acclimatization period (2a), a trend favouring the test and positive control groups for both endpoints (Schiff sensitivity score and tactile threshold) compared with the negative control was observed at Weeks 4 and 8; however, none of these differences were statistically significant.

### Safety

A total of 32 (16.2%) subjects reported 42 treatment-emergent AEs (TEAEs) during the study (Table S3): 38 TEAEs were resolved by the end of the study and 4 TEAEs were reported in 4 subjects (1 event in each subject) as ongoing at the end of the study (3 TEAEs were oral and 1 non-oral [chronic pharyngitis]). The 4 subjects reporting these 4 TEAEs were lost to follow-up. Among the 42 TEAEs, 33 were oral (Table S3), of which 30 were resolved by the end of the study. Three oral TEAEs were ongoing at the end of the study (1 mild: broken tooth; 2 moderate: 2 broken teeth). A total of 9 TEAEs were non-oral and reported in 7 (3.6%) subjects. Three (4.5%) subjects reported 4 TEAEs in the test product group and 4 (6.3%) subjects reported 5 TEAEs in the positive control dentifrice group (Table S3).

Additionally, there were 2 serious AEs (gallstone pancreatitis and gallstone with acute cholecystitis) reported for 1 subject in the test product group; no serious AEs were reported in the negative control dentifrice and positive control dentifrice groups. None of the TEAEs were considered treatment-related and no AEs related to Covid-19 were reported during this study. There were no deaths and no TEAEs reported that led to study or product discontinuation.

## Discussion

DH is a commonly experienced oral health problem with increasing prevalence, particularly in patients aged 30–40 years old, due to factors such as diet, brushing habit and lifestyle [1, 2]. A higher prevalence of DH is typically reported in women; whilst there is no known evidence demonstrating that gender may influence DH, a 2009 National DH Survey conducted in rural China showed that a greater proportion of those diagnosed with DH were female (35.8%), in comparison to a lower proportion that were male (23.4%) [37]. This might explain the high female: male ratio of 3:1 seen in this study, where the majority (90.3%) of the population were female.

This 8-week randomized controlled trial investigated the efficacy of a SnF_2_ dentifrice (test product) in comparison with a regular fluoride dentifrice (negative control, 1150 ppm NaF) in the relief of DH in a Chinese population. A positive control dentifrice with proven long-term DH efficacy in China was included in this study, as there is previous clinical evidence on DH relief in a Chinese population [30, 31]. All treatment groups showed improvements in DH relief, including the negative control, with a statistically significant decrease (p < 0.0001) in the change from baseline in Schiff sensitivity score and tactile threshold at all timepoints. The negative control used in this study was a commercially available regular fluoride toothpaste with no known anti-sensitivity benefits; however, a large reduction in the mean Schiff sensitivity score from baseline (mean = 2.43) was observed at Week 8 (mean change = –1.14).

This unexpected performance of the negative control dentifrice led to no statistically significant differences in the adjusted mean Schiff sensitivity score at Week 8 only (p = 0.2639), or in the adjusted mean tactile threshold (p = 0.8130) between the SnF_2_ test dentifrice vs. the negative control dentifrice and positive control vs. negative control groups at all timepoints; therefore, the primary and secondary objectives were not met. These results are contradictory with the extensive body of evidence of worldwide clinical studies that prove the efficacy of SnF_2_ in improving DH symptoms [17, 38, 39].

Generally, DH studies can be difficult to conduct due to the challenge of defining pain and the subjectivity of pain or discomfort associated with DH in each participant [17, 40]. Furthermore, clinical trials evaluating clinical endpoints relating to pain and specifically to DH can be prone to ‘placebo effects’, as well as the Hawthorne effect, characterized by an improvement in symptoms simply as a function of clinical study participation [41, 42]. Results from our study clearly showed a strong placebo effect; therefore, the data were further investigated in a post-hoc analysis to understand factors that could have caused the strong placebo effect observed.

Due to the Covid-19 outbreak in China in 2021, the clinical study was put on hold, resulting in one-third of the study population having an extended acclimatization period of 2.5 months (vs. 4 weeks as per protocol). Furthermore, in depth evaluation of the data showed subjects with non-stable DH condition (1b) had a notably higher mean baseline Schiff sensitivity score compared with those with stable DH condition (1a). The aim of the post-hoc analysis was then to explore the impact of DH fluctuations between screening and baseline and the extended acclimatization period on the placebo/Hawthorne effect observed in the tactile threshold and Schiff sensitivity score results. The post-hoc analysis indicated that DH stability and an extended acclimatization period may have had an impact on the magnitude of the measured DH effect observed in the tactile threshold and Schiff sensitivity score results. The data showed greater changes from baseline in both tactile threshold (g) and Schiff sensitivity score in the negative control group for those subjects with an extended acclimatization period and in subjects with non-stable DH condition, leading to the hypothesis that both factors could have impacted the results. These observations add information to the growing field of DH research to better understand why the placebo effect is a common phenomenon in DH studies, as well as give useful insights for future clinical study design. However, it is important to note that the results of this post-hoc analysis have limitations due to the small sample sizes and the exploratory nature of this analysis; as such, results should be interpreted with caution.

Another possible limitation is that long-term consumption of acidic foods, such as carbonated beverages and citrus fruits, might aggravate DH. Thus, subjects' dietary preferences might lead to biased results of DH tests, and instruction on acidic food intakes should be provided to subjects. A final limitation may be that oral hygiene condition were not standardized or controlled. Further studies may need to consider being more prescriptive about oral hygiene status.

Nevertheless, the beneficial effect of the active ingredients used in this study was confirmed by the observed improvements in OHRQoL. The DHEQ-15 responses were useful to gain a greater understanding of OHRQoL and the effect of DH on participants’ day-to-day lives, in particular the impact on daily activities (restriction), modification of habits (adaptation), interactions with other people (social impact), emotions (emotional impact), and health and/or age perception (identity). Validation studies on DHEQ, and its use in the Chinese population, showed high internal reliability, test–retest reliability and criterion validity in DH studies [25, 26, 43]. In this study, the DHEQ total score and all domains demonstrated a statistically significant improvement (from baseline) in OHRQoL (p < 0.0001) for all treatment groups at each timepoint, with a trend of reduction over time, favouring both the test and positive control vs. negative control dentifrice. However, no significant differences between the test product or the positive vs. negative control dentifrice were observed.

## Conclusions

Overall, all product groups demonstrated statistically significant improvements (change from baseline) in Schiff sensitivity score, tactile threshold and OHRQoL. However, due to the strong placebo effect observed in this study, there was no statistically significant difference between the three products. This study highlights the inherent complexity of measuring and recording DH, especially given the subjective nature of perceived pain. Further studies could include more detailed patient-reported outcomes.

### Supplementary Information

Below is the link to the electronic supplementary material.

Supplementary file1 (DOCX 14 KB)
