# Supplementary material for: Efficacy of a stannous fluoride dentifrice for relieving dentinal hypersensitivity in Chinese population: an 8-week randomized clinical trial
Source: Clin Oral Investig. 2024 Mar 26;28(4):230. doi: 10.1007/s00784-024-05610-9 (PMC10965716; doi:10.1007/s00784-024-05610-9)
Supplement: Supplementary file 1 — Supplementary file1 (DOCX 14 KB) [file 784_2024_5610_MOESM1_ESM.docx]

**Supplementary material**

**Efficacy of a Stannous Fluoride Dentifrice for relieving Dentinal Hypersensitivity in Chinese Population: an 8-week randomized clinical trial**

**Authors:** Rui Li^1^, Wenjie Yang^1^, Roberta Grimaldi^2^, Peter Zeng^3^, Gary Smith^2^ and Xi Chen^1^

**Affiliations:** ^1^Department of Preventive Dentistry, Shanghai Ninth People’s Hospital, Shanghai, China; Shanghai Jiao Tong University School of Medicine; College of Stomatology, Shanghai Jiao Tong University; National Center for Stomatology; National Clinical Research Center for Oral Diseases; Shanghai Key Laboratory of Stomatology; Shanghai Research Institute of Stomatology, Shanghai, China; ^2^Haleon (formerly known as GSK Consumer healthcare), Weybridge, UK; ^3^Haleon (formerly known as GSK Consumer healthcare), Shanghai, China.

**Corresponding author:** Dr Xi Chen, Shanghai Ninth People’s Hospital, No. 639 Zhizaoju Road, Shanghai, China – 200011. Email: [chenx1853@sh9hospital.org.cn](mailto:chenx1853@sh9hospital.org.cn). Phone number: +86 21 53315327.

# Supplemental methods

## Number of brushings and brushing compliance

Subject exposure to the study product was reported as study product compliance and the number of brushings. Compliance data with use of the study product were summarized for the mITT population and assessed by both the number of brushings and the study dentifrice weight. Overall, a total of 60 evaluable subjects per group meant the study had 90% power to detect a mean difference of 0.3 (standard deviation [SD] = 0.501) in change from baseline in Schiff sensitivity score after 8 weeks of treatment. The difference of 0.3 represented roughly a 15% difference between treatment groups.

The number of brushings was defined as the date of visit (date of Visit_n_ – the date of Visit_n-1_ + 1), multiplied by two, minus the number of missing brushings plus the number of additional brushings by the visits (date of Visit 4 – date of Visit 2 +1). The visits were then multiplied by two, minus the number of missing brushings plus the number of additional brushings, by overall. Brushing compliance (%) was defined as 100 multiplied by the number of brushings/expected number of brushings. The expected number of brushings was defined as the date of visits (date of Visit_n_ – date of Visit_n-1_ + 1), multiplied by 2, by overall.

## Change from baseline in Schiff sensitivity score and tactile score

Schiff sensitivity score was calculated as the average score of the two test teeth for secondary efficacy variables. Descriptive statistics (n, missing, mean, SD, SE, median, minimum and maximum) were presented for tactile threshold (g) and calculated as the average score of the two test teeth at each assessment timepoint together with the change from baseline for all subjects in the mITT population by study product.

The tactile score was derived in the same manner as for the Schiff score (i.e. derived as the average score of the two test teeth). The change from baseline was derived from the individual teeth first before calculating the average change of the two test teeth. The tooth recorded as >20 g (for screening and baseline) or >80 g (for post-baseline) was rounded up to the next increment of 10 g for the calculation of average tactile threshold (g).
